# Synthesis and Characterization of New Thiazolidinones Containing Coumarin Moieties and Their Antibacterial and Antioxidant Activities

**DOI:** 10.3390/molecules17089321

**Published:** 2012-08-03

**Authors:** Naceur Hamdi, Abdullah Sulaiman Al-Ayed, Ridha Ben Said, Alary Fabienne

**Affiliations:** 1 College of Science and Arts at Ar-Rass, Qassim University, P.O. Box 53, Saudi Arabia; 2 Laboratoire de chimie et physique quantiques, Université Paul Sabatier-Bât. 3R1b4, 118 route de Narbonne, 31062 Toulouse Cedex 09, France

**Keywords:** coumarin, ethyl bromoacetate, thiazolidinones, DFT studies, antibacterial activities

## Abstract

New coumarin derivatives, namely (2-(4-methyl-2-oxo-*2H*-chromen-7-yloxy)-*N*-(4-oxo-2-phenylthiazolidin-3-yl)acetamide, *N*-(2-(3-methoxyphenyl)-4-oxothiazolidin-3-yl)-2-(4-methyl-2-oxo-*2H*-chromen-7-yloxy)acetamide, 2-(4-methyl-2-oxo-*2H*-chromen-7-yloxy)-*N*-(4-oxo-2-(2,3,4trimethoxyphenyl)thiazolidin-3-yl)acetamide and *N*-(2-(4-bromophenyl)-4-oxothiazolidin-3-yl)-2-(4-methyl-2-oxo-*2H*-chromen-7-yloxy)acetamide) were synthesized starting from 4-methyl-7-hydroxycoumarin. The structures of the obtained compounds were confirmed by analytical IR and NMR spectra to elucidate the different positions of protons and carbons and as well as theoretical studies (DFT/B3LYP). The new compounds were screened for antibacterial activity. Most of them are more active against *E. coli S. aureus* and *B. subtilis* than standard references.

## 1. Introduction

Small ring heterocycles containing nitrogen and sulfur have been under investigation for a long time because of their important medicinal properties. Among the wide range of heterocycles explored to develop pharmaceutically important molecules, thiazoles have played an important role in medicinal chemistry. A survey of literature has shown that compounds having a thiazole nucleus possess a broad range of biological activities such as anti-inflammatory [1], antibacterial [2] and antifungal properties [3]. Among these type of molecules 4-thiazolidinones have shown to have various important biological activities such as antibacterial, antifungal, antiviral, diuretic, antituberculostatic, anti-HIV, antihistaminic, anticancer, anticonvulsant, anti-inflammatory and analgesic properties [4,5,6,7,8,9,10,11]. Recently, a study reported the synthesis, chemical and wide rang biological properties of a series of 4-thiazolidinone molecules [12,13,14,15,16,17,18]. Some of these compounds showed moderate to good biological properties. The observed interesting biological properties of this class of compounds impelled us to synthesize new examples with possible improved biological properties with applicative possibilities.

In the current study we aimed to synthesize some new coumarins derived from umbelliferone (7-hydroxycoumarin), with predictable biological activities. The chemical structures of the synthesized compounds were proven by IR, NMR spectra and elemental analysis data.

## 2. Results and Discussion

The starting compound (7-hydroxy-2-oxo-*2H*-chromen-4-yl)-acetic acid methyl ester (**2**) was prepared in 92% yield by esterification of 7-hydroxy-4-methylcoumarin (**1**). Hydrazinolysis of **2** with 86% hydrazine hydrate in ethanol at reflux for 4 h afforded hydrazide **3** in good yield. The FT-IR spectra of carbohydrazide **3** showed absorption bands at 3317 cm^−1^, possibly due to the hydrazide NH-NH_2_, 3269 cm^−1^ (aromatic CH), 1711 cm^−1^ (-C=O carbonyl stretching) and in the range 1621–1640 cm^−1^ (-CO-NH-NH_2_ groups). The ^1^H-NMR spectrum exhibited a singlet due to the -CO-N*H*-NH_2_ proton at δ 4.34 ppm. Methylene protons (–OCH_2_) resonate as singlets at 4.94 ppm.

Refluxing **3** and aromatic aldehydes with a catalytic amount of glacial acetic acid in absolute ethanol for 4 h afforded the new series of coumarin *N′*-benzylidene-2-(4-methyl-2-oxo-*2H*-chromen-7-yloxy) acetohydrazide Schiff bases **4a**–**d**. The structures of compounds **4a**–**d** were inferred from their analytical and spectroscopic properties. Thus the IR spectrum of compound **4a** showed characteristic bands in the range 1610 cm^–1^ (CONH), at 1681 cm^–1^ (C=O, lactone), and at 1258 cm^–1^ (-HC=N- azomethine). The ^1^H-NMR spectrum did not only show the absence of NH_2_ protons at 3.38, but also the presence of the N=CH proton at 8.24 ppm.

**Scheme 1:**
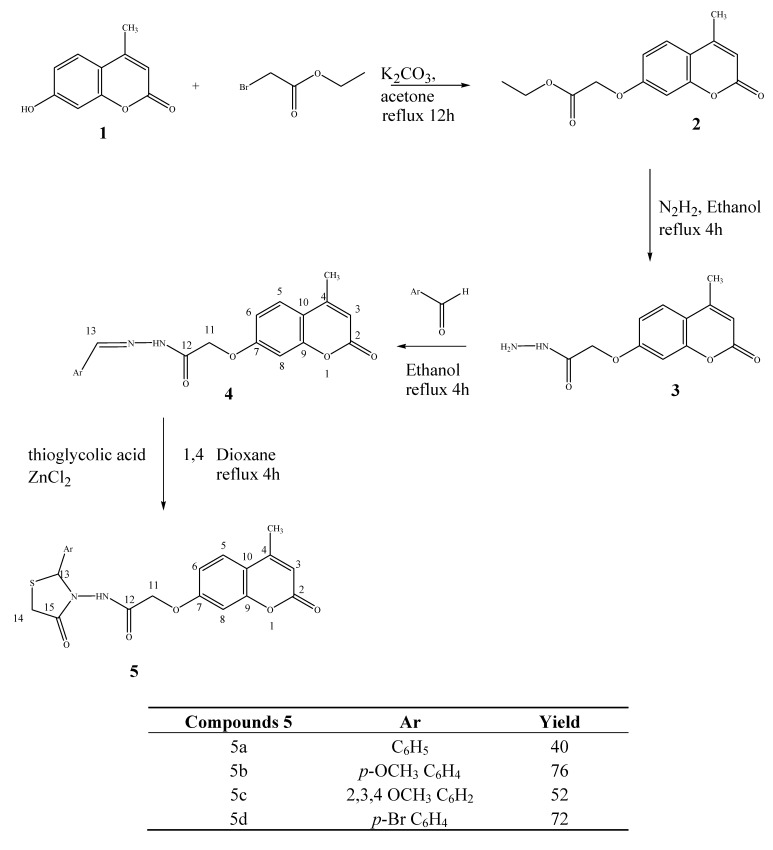
Synthesis of 2-(4-methyl-2-oxo-*2H*-chromen-7-yloxy)-*N*-(4-oxo-2-arylthiazolidin-3-yl) acetamide 5.

*N*-(2-aryl-4-oxo-thiazolidin-3-yl)-2-(4-(2-aryl-4-oxo-thiazolidin-3-ylcarbamoyl)-methyl)-2-oxo-*2H*-chromen-7-yloxy)-acetamides **5a**–**d** were obtained by reaction of the compounds **4a**–**d** with thioglycolic acid in refluxing 1,4-dioxane for 6–8 h in the presence of anhydrous ZnCl_2_ (Scheme 1).

**Figure 1:**
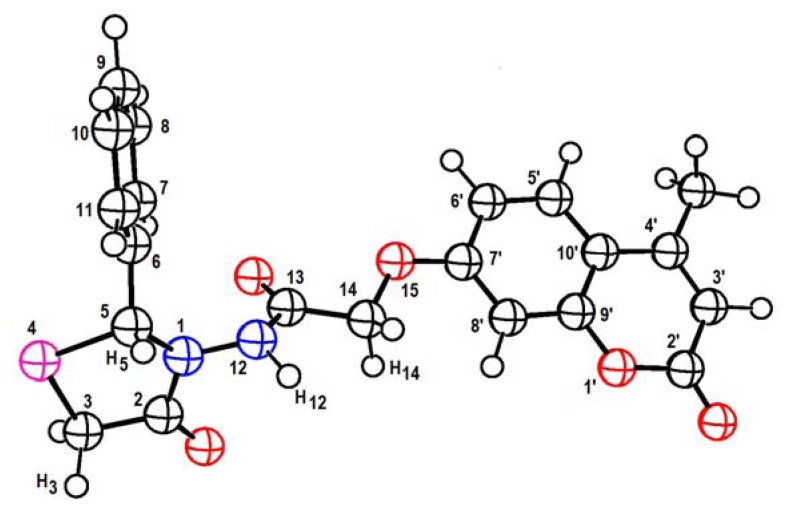
Optimized geometry for **5a**.

The IR spectrum of **5b** showed a characteristic band at 1728 cm^−1^ that supports the presence in the molecule of a thiazolidinone C=O group. The ^1^H-NMR spectrum of **5b** displayed signals between 7.30–7.60 δ ppm for aromatic protons and a doublet at 4.84 ppm ascribable to the thiazolidinones CH_2_ protons. The proposed **5a**–**d** structures were evaluated by using a DFT/B3LYP approach implemented in the Gaussian 09 series programs [19]. The B3LYP hybrid functional has been used in describing potential energy surfaces (PES). The geometries of the compounds were fully optimized using analytic gradients. The harmonic vibrational frequencies of the stationary points of the PES have been calculated at the same level of theory in order to identify the local minima as well as to estimate the corresponding zero point vibrational energy (ZPE) [20]. For each atom no pseudopotential are used. A Def2-SVP EMSL Basis Set Exchange was employed for each atom [21]. Values of selected geometrical parameters are listed in Table 1 and optimized geometry for **5a** is depicted in Figure 1.

**Table 1:** DFT/B3LYP optimized geometrical parameters ^a^ for **5a**–**d**.

Compounds	5a	5b	5c	5d
1-2	1,385	1.384	1.383	1.386
1-12	1.374	1.374	1.372	1.374
1-5	1.463	1.465	1.462	1.462
2-3	1.520	1.521	1.520	1.520
3-4	1.829	1.829	1.826	1.829
4-5	1.863	1.864	1.866	1.863
5-6	1.510	1.506	1.509	1.510
12-13	1.394	1.393	1.389	1.394
13-14	1,530	1.530	1.531	1.529
14-15	1.401	1.401	1.402	1.401
15-7′	1.352	1.352	1.352	1.353
1′-2′	1.396	1.396	1.396	1.397
1′-9′	1.354	1.354	1.354	1.354
2′-3′	1.457	1.457	1.457	1.457
3′-4′	1.362	1.362	1.362	1.362
4′-10′	1.454	1.454	1.454	1.454
5-H5	1.105	1.106	1.101	1.105
12-H12	1.020	1.020	1.020	1.020
14-H14	1.107	1.108	1.108	1.108
1-2-3	111.1	111.0	110.7	111.1
2-3-4	107.8	107.8	107.6	107.8
3-4-5	92.9	92.8	93.0	92.9
4-5-1	103.4	103.2	103.4	103.5
1-5-6	115.5	115.6	115.6	115.5
1-5-H5	109.5	109.5	108.4	109.6
2-1-12	118.1	118.2	119.5	118.1
1-12-H12	114.9	114.9	114.7	114.8
1-12-13	119,0	118.9	121.2	119.0
12-13-14	111.9	111.9	111.2	112.0
13-14-15	108.3	108.3	108.9	108.4
1′-2′-3′	115.4	115.4	115.4	115.4
2′-3′-4′	123.3	123.3	123.3	123.3
3′-4′-10′	118.6	118.6	118.5	118.5
4′-10′-9′	118.0	118.0	117.9	118.0
1-2-3-4	6.1	7.5	−13.9	6.5
2-3-4-5	−15.4	−17.0	18.6	−15.8
3-4-5-1	19.9	21.1	−17.9	20.0
H5-5-6-11	−12.6	−11.8	−0.7	−12.8
3-4-5-6	144.3	145.8	109.4	144.5
1-5-6-7	−136.1	−135.1	−122.1	−136.4
2-3-5-12	1.0	1.1	−0.9	−11.6
2-5-7′-6′	113.0	114.1	109.1	118.2
12-13-14-15	−158.1	−157.1	−161.7	−155.2
13-14-15-7′	−175.5	−174.8	−177.7	−174.9
14-15-7′-6′	178.5	178.2	176.8	179.2
7′-8′-9′-10′	0.0	0.0	0.4	0.1
10′-9′-1′-2′	0.0	0.1	0.0	0.0
1′-2′-3′-4′	0.1	0.2	0.6	0.1

^a^ Distances are in A˚ and angles in degrees.

Some conclusions can be extracted from the results obtained. The calculated values for length and angles are similar for compounds **5a**–**d**. This fact suggests that the R group has no influence on interatomic distances and angles. Additionally the dihedral angles 1-2-3-4, 2-3-4-5 and 3-4-5-1 indicate that the thiazolidinone ring in the compounds is not in a plane and that fact is not dependent on the R group. The plane constituted by the thia-ring is perpendicular to that of the coumarin group (for exemple the angle 2-5-6′-7′ = 113.0 in **5a**). It is important to point out that the **5c** thia-ring displays a different orientation that those for the other calculated compounds. Important information on the chemical groups present in compounds **5a**–**d** can be obtained from their calculated vibrational spectra. As example, intensities and their harmonic wave numbers calculated with the method DFT/B3LYP and the corresponding experimental values of the compound **5c** are grouped in Table 2.

**Table 2:** Theoretical and experimental vibrational frequencies (cm^−1^) and theoretical infrared intensities (km mol^−1^) of **5c**.

	Experimental		Theoretical	
Functional group	Frequencies	Intensities	Frequencies	Intensities
CO thiaz.	1666	strong	1831	328
CO lactone	1682	strong very	1862	652
CO amide	1712	mean	1871	130
NH	3313	weak	3513	23

The values of the theoretical IR frequencies are in agreement with the experimental ones. The largest shifting is less than 10% that could be due to the approximations used in the DFT/B3LYP method; consequently the proposed structures were supported by theoretical calculations.

The NBO analysis allows the determination of the atomic natural charges. Table 3 reports the natural charges of the acid protons in compound **5c** calculated at DFT/B3LYP level of theory. We find that proton H_12_ carried by the nitrogen was the most acidic.

**Table 3:** NBO charge of the acidic protons of the compound **5c** calculated at the DFT/B_3_LYP level of theory.

Hi	Charge
H_3_	0.25
H_5_	0.22
H_12_	0.39
H_14_	0.22

### 2.1. Antioxidant Activities

The imbalance between reactive oxygen species (ROS) and antioxidant defence mechanisms leads to oxidative modification in cellular membrane or intracellular molecules. Phytochemicals like phenolics, commonly found in plants, could constitute strong natural antioxidants and could play an important role in health care system. 

**Figure 2:**
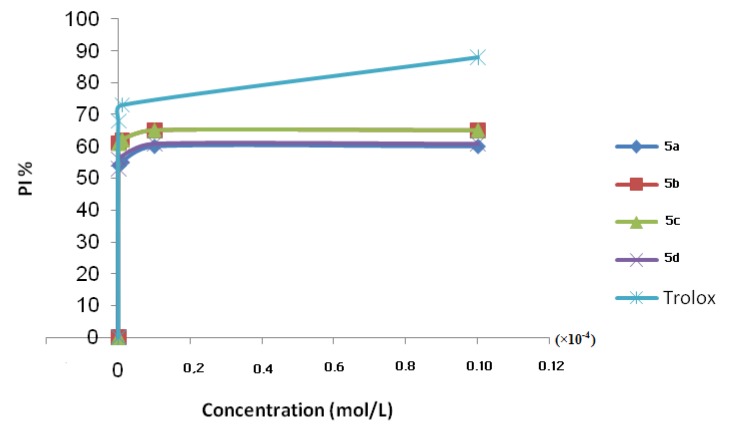
Scavenging effect on 1,1-diphenyl-2-picrylhydrazyl (DPPH) radical of compound **5**.

The effect of the different synthetic compounds on DPPH radical scavenging was compared to those of Trolox, using as positive control, and appreciated by the determination of the IC_50_ values. The results are shown in Figure 2 and listed in Table 4.

**Table 4:** Values of IC50 exhibited by coumarinic derivatives **5a**–**d**.

Compounds 5	IC_50_ (10^−9^ mol/L)
**5a**	92.60
**5b**	82.00
**5c**	8.62
**5d**	9.43
Trolox	7.35

As shown in Figure 2, DPPH test revealed that increase in compounds concentration resulted in increase in free radical-scavenging activity in a dose dependent manner. Based on the IC_50_ values, the most active compound is **5c**.

### 2.2. ABTS Radical Cation Decolourization Assay

**Figure 3:**
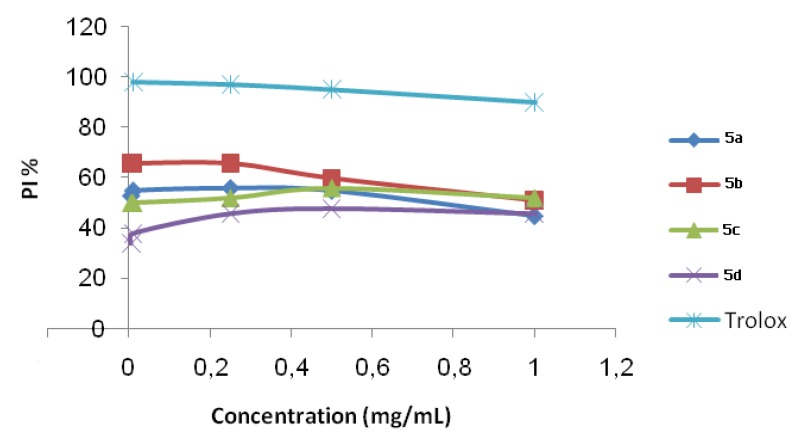
Scavenging ability on ABTS radical of compounds **5**.

As shown for DPPH scavenging, the obtained results indicate the higher capacity of **5a**–**d** to quench ABTS as compared to the synthetic antioxidant Trolox (Figure 3 and Table 5).

**Table 5:** Values of IC50 exhibited by coumarinic derivatives **5a**–**d**.

Compounds 5	IC_50_ (10^−9^ mol/L)
**5a**	92.60
**5b**	82.00
**5c**	8.62
**5d**	9.43
Trolox	7.35

### 2.3. Antibacterial Activity

The antibacterial activity of the tested compounds against a series of bacteria and fungi is shown in Table 6.

**Table 6:** Antibacterial activity against bacteria of **5a**–**d**, inhibition zone expressed in mm.

Organism	Compounds				Standard-drug
	5a	5b	5c	5d	ampicillin
*E. coli* **ATCC1225**	19	19	19	19	16
*P. vulgaris*	20	20	20	20	22
*B. megaterium*	17	17	17	17	23
*S. aureus* **ATCC2353**	18	18	18	18	25

All the compounds **5** displayed maximum activity against *P. vulgaris*. The compound **5b** is highly active against *E. coli*. The compounds **5b** and **5d** also showed very good activity against *B. megaterium*, while compounds **5a** and **5c** showed good activity against *S. aureus*.

Our results also showed that the purified compounds had great potential for antibacterial activity against a panel of microorganisms. There is evidence in the literature that Gram-positive bacteria are more sensitive to plant extracts and essential oil than Gram-negative bacteria [22]. On the basis of inhibition zone diameters values *P. vulgaris* was more sensitive to the purified compounds than the other Gram positive bacteria. The observed differences in the inhibition zones within pathogenic bacteria could be probably due to cell membrane permeability or other genetic factors.

## 3. Experimental

### 3.1. General

All reagents were obtained from commercial sources. Solvents were either commercially obtained as analytical grade or freshly distilled prior to use. Analytical thin layer chromatography was carried out on precoated silica gel 60F254 plates using either UV absorption or iodine staining for visualization. Column chromatography was carried out using 100–200 mesh silica gel. ^1^H-NMR and ^13^C{^1^H}-NMR spectra were recorded on a Bruker DRX 300 MHz instrument (300/75 MHz); chemical shift values are reported relative to tetramethylsilane as internal standard. The IR spectra were recorded on a Thermo Mattson IR300 and Nicolet 510 PET spectrometers. Elemental microanalysis was carried out using a model 5500-Carlo Erba C.H.N.S.O elemental analyzer instrument.

*General procedure for the preparation of ethyl 2-((4-methyl-2-oxo-2H-chromen-7-yl)oxy)acetate* (**2**)*.* A mixture of 7-hydroxy-4-methylcoumarin (5.6 mmol), anhydrous potassium carbonate (5.6 mmol) and ethyl bromoacetate (5.6 mmol) in dry acetone (10 mL) was refluxed with continuous stirring for 12 h. After filtration, the solution was concentrated under reduced pressure, vacuum dried and the solid product was recrystallized from ethanol. m.p. 185–186 °C, yield 92%; IR: ν_max_ CO lactone: 1,714 cm^−1^, NH: 3,435 cm^−1^, CO (ketone): 1,606 cm^−1^; ^1^H-NMR: δ 7.76 (d, *J*H_5.6_ = 14.6 Hz, 1H, H-5), 7.04 (d, *J*H_5.6_ = 14.6 Hz, 1H, H-6), 7.02 (s, 1H, H-8), 6.34 (s, 1H, H-3), 4.92 (s, 2H, -OCH_2_), 4.19 (q, *J*H_2_-H_3_ = 10.6 Hz, 2H, CH_2_, -CH_2_CH_3_), 4.02 (s, 2H, CH_2_), 1.22 (t, *J*H_2_-H_3_ = 10.6 Hz, 3H, CH_3_, -CH_2_CH_3_); ^13^C{^1^H}- NMR: δ 14.2 (CH_2_CH_3_), 34.8 (CH_2_CO), 61.3 (CH_2_CH_3_), 65.5 (COCH_2_O), 109.6 (C-8), 112.8 (C-6), 113.8 (C-3), 114.8 (C-10), 128.3 (C-5), 151.2 (C-9), 155.2 (C-4), 160.3 (C-7), 160.9 (C-2), 168.9 (CO-O), 169.3 (C-CO-C). Found, %: C, 64.12; H, 5.2; O, 30.40. C_14_H_14_O_5_. Calculated, %: C, 64.12; H, 5.38; O, 30.50.

*2-(4-Methyl-2-oxo-2H-chromen-7-yloxy)acetohydrazide* (**3**). To a solution of ethanol (15 mL) and hydrazine hydrate (10 mmol), ethyl 2-(4-methyl-2-oxo-*2H*-chromen-7-yloxy) acetate (**2**, 10 mmol) was added, and the mixture was refluxed for 4 h. The product precipitated and was collected by suction filtration, washed with methanol and recrystallized from dil. Acetic acid. m.p. = 300 °C, yield 70%; IR: ν_max_ CO lactone: 1,681 cm^−1^, CONH (amide): 1,612 cm^−1^, CN: 1,271 cm^−1^; ^1^H-NMR: δ 9.41 (s, 1H, NH), 7.76 (d, *J*H_5.6_ = 14.6 Hz, 1H, H-5), 7.04 (d, *J*H_5.6_ = 14.6 Hz, 1H, H-6), 7.02 (s, 1H, H-8), 6.34 (s, 1H, H-3), 4.94 (s, 2H, -OCH_2_), 4.34 (s, 2H, NH_2_). ^13^C{^1^H}-NMR: δ 45.8 (CH_2_), 68.9 (CH_2_O-), 108.0 (C-8), 111.8 (C-6), 112.9 (C-3), 114.1 (C-10), 128.3 (C-5), 152.2 (C-9), 155.2 (C-4), 160.4 (C-7), 160.9 (C-2), 166.8 (COCH_2_O), 169.6 (COCH_2_). Found, %: C, 58.1; H, 4.82; N, 11.1; O, 25.70 C_12_H_12_O_4_N_2_. Calculated, %: C, 58.06; H, 4.87; N, 11.29; O, 25.78.

*General procedure for the preparation of N′-benzylidene-2-(4-methyl-2-oxo-2H-chromen-7-yloxy) acetohydrazide* (**4**). A mixture of 2-(4-methyl-2-oxo-*2H*-chromen-7-yloxy)acetohydrazide (**3**, 3.06 g, 0.01 mol) and appropriate aromatic aldehyde (**Ar/a-d**, 0.01 mol) was refluxed in absolute ethanol (30 mL) in the presence of a catalytic amount of glacial acetic for 4 h. The reaction mixture was cooled; the solid separated was filtered and recrystallized from methanol to give compounds **4a**–**d**.

*N'-Benzylidene-2-((4-methyl-2-oxo-2H-chromen-7-yl)oxy)acetohydrazide* (**4a**). m.p. 268–269 °C; yield 74%; IR: ν_max_ CO lactone: 1681 cm^−1^, CONH (amide): 1610 cm^−1^, C=N: 1258 cm^−1^; ^1^H-NMR: δ 8.30 (s, 1H, HC=N-), 8.02 (s, 1H, NH), 7.76 (d, *J*H_5.6_ = 15.6 Hz, 1H, H-5), 7.72–7.31 (m, 10H, arom.), 7.04 (d, *J*H_5.6_ = 15.6 Hz, 1H, H-6), 7.02 (s, 1H, H-8), 6.34 (s, 1H, H-3), 4.83 (s, 2H, -OCH_2_), ^13^C{^1^H}-NMR: δ 45.7 (CH_2_), 69.2 (CH_2_O-), 108.1 (C-8), 111.6 (C-6), 112.5 (C-3), 114.4 (C-10), 127.9 (C-5), 128.4 (C-3,5, Ar-), 129.0 (C-2,6, Ar-), 131.4 (C-4, Ar-), 133.9 (C-1, Ar-), 143.6 (N=CH-), 151.8 (C-9), 155.2 (C-4), 160.4 (C-7), 160.9 (C-2), 166.9 (COCH_2_O), 170.0 (CONH-). Found, %: C, 67.75; H, 4.8; N, 8.2; O, 19.1. C_19_H_16_O_4_N_2_. Calculated, %: C, 67.85; H, 4.79; N, 8.33; O, 19.03.

*N′-(3-Methoxybenzylidene)-2-((4-methyl-2-oxo-2H-chromen-7-yl)oxy)acetohydrazide* (**4b**). m.p. 225–226 °C, yield (76%); IR: ν_max_ CO lactone:1,681 cm^−1^,CONH (amide): 1,614 cm^−1^, C=N: 1,255 cm^−1^; ^1^H-NMR: δ 8.73 (s, 1H, -HC=N-), 8.45 (s, 1H, NH), 7.72 (d, JH_5.6_ = 15.5 Hz, 1H, H-5), 7.60–7.30 (m, 8H, arom.), 3.65 (s, 3H, OCH_3_) ,7.04 (d, JH_5.6_ = 15.4 Hz ,1H, H-6), 7.02 (s, 1H, H-8), 6.34 (s, 1H, H-3), 4.84 (s, 2H, -OCH_2_), ^13^C{^1^H} NMR: δ 45.6 (CH_2_), 68.9 (CH_2_O-), 107.8 (C-8), 111.5(C-6), 112.7 (C-3), 113.9 (C-10), 127.8 (C-5), 129.4 (C-3, Ar-), 130.6 (C-6, Ar-), 132.7 (C-4, Ar-),133.8 (C-1, Ar-), 134.3 (C-2, Ar-), 143.5 (N=CH-), 151.7 (C-9), 155.0 (C-4), 160.3 (C-7), 160.9 (C-2),166.4 (COCH_2_O), 169.9 (CONH-). Found, %: C, 65.5; H, 4.80; N, 7.6; O, 21.8. C_20_H_18_O_5_N_2_. Calculated, %: C, 65.57; H, 4.95; N, 7.65; O, 21.84.

*N′-(2.3.4-Trimethoxybenzylidene)-2-((4-methyl-2-oxo-2H-chromen-7-yl)oxy)acetohydrazide* (**4c**). m.p. 259–261 °C, yield 72%; IR: ν_max_ CO lactone: 1,681 cm^−1^, CONH (amide): ,1618 cm^−1^, C=N: 1,281 cm^−1^. ^1^H-NMR: δ 8.31 (s, 1H, -HC=N-), 7.76 (d, *J*H_5.6_ = 15.5Hz, 1H, H-5), 7.67–7.30 (m, 8H, arom.), 7.04 (d, *J*H_5.6_ = 15.6 Hz ,1H, H-6), 7.02 (s, 1H, H-8), 6.34 (s, 1H, H-3), 3.78 (s, 9H, 2,3,4 OCH_3_), 4.84 (s,2H, -OCH_2_), ^13^C{^1^H}-NMR: δ 45.5 (CH_2_), 69.1 (CH_2_O-), 107.9 (C-8), 111.4 (C-6),112.5 (C-3), 113.4 (C-10), 127.3 (C-6, Ar-), 127.9 (C-5), 129.3 (C-2, Ar-), 130.3 (C-5, Ar-), 131.2 (C- 4, Ar-), 135.2 (C-1, Ar-), 134.4 (C-3, Ar-), 143.4 (N=CH-), 151.6 (C-9), 155.2 (C-4), 160.6 (C-7), 160.9 (C-2), 166.7 (COCH_2_O), 169.8 (CONH-). Found, %: C, 61.9; H, 5.1; N, 6.5; O, 26.3. C_22_H_22_O_7_N_2_. Calculated, %: C, 61.97; H, 5.20; N, 6.57; O, 26.26.

*N′-(4-Bromobenzylidene)-2-((4-methyl-2-oxo-2H-chromen-7-yl)oxy)acetohydrazide* (**4d**). m.p. 273–275 °C, yield 52%; IR: ν_max_ CO lactone: 1,681 cm^−1^, CONH (amide): 1,612 cm^−1^, ^1^H-NMR: 8.30 (s, 1H, -HC=N-), 8.23 (s, 1H, NH), 7.76 (d, *J*H_5.6_ = 15.5 Hz, 1H, H-5), 7.61–7.30 (m, 6H, arom.), 7.04 (d, *J*H_5.6_ = 15.5 Hz, 1H, H-6), 7.02 (s, 1H, H-8), 6.34 (s, 1H, H-3), 4.82 (s, 2H, -OCH_2_), ^13^C{^1^H}- NMR: δ 45.6 (CH_2_), 69.2 (CH_2_O-), 103.8 (C-3, Ar-), 107.6 (C-8), 108.7 (C-5, Ar-),111.3 (C-6), 112.7 (C-3), 113.4 (C-10), 127.2 (C-6, Ar-), 127.9 (C-5), 135.2 (C-1, Ar-), 143.0 (N=CH-), 151.2 (C-9), 155.1 (C-4), 160.5 (C-7), 160.9 (C-2), 162.4 (C-2, Ar-), 162.6 (C-4, Ar-), 166.5 (COCH_2_O), 169.4 (CONH-). Found, %: C, 54.8; H, 3.6; Br, 19.1; N, 6.7; O, 15.4. C_19_H_15_O_4_N_2_. Calculated, %: C, 54.96; H, 3.64; Br, 19.24; N, 6.75; O, 15.41.

*General procedure for the preparation of 2-(4-methyl-2-oxo-2H-chromen-7-yloxy)-N-(4-oxo-2 arylthiazolidin-3-yl) acetamide* (**5**). A mixture of *N′*-benzylidene-2-(4-methyl-2-oxo-*2H*-chromen-7-yloxy) acetohydrazide (**4**, 0.01 mol) and mercaptoacetic acid (1.82 g, 0.02 mol) in 1,4-dioxane (30 mL) containing a pinch of anhydrous ZnCl_2_ was refluxed for 6–8 h. The reaction mixture was cooled and poured onto crushed ice. The solid thus obtained was filtered, washed with water and recrystallized from DMF yielding **5a**–**d**.

*2-(4-Methyl-2-oxo-2H-chromen-7-yloxy)-N-(4-oxo-2-phenylthiazolidin-3-yl)acetamide* (**5a**). m.p. 202–204 °C, yield 40%; IR: ν_max_ CO lactone: 1,681 cm^−1^, CONH (amide): 1,615 cm^−1^; ^1^H-NMR: 2.15 (s, 3H), 8.12 (s, 1H, -NH), 7.76 (d, *J*H_5.6_ = 10.6 Hz, 1H, H-5), 7.71–7.23 (m, 10H, arom.), 7.04 (d, *J*H_5.6_ = 10.6 Hz, 1H, H-6), 7.02 (s, 1H, H-8), 6.34 (s, 1H, H-3), 5.92 (s, 1H, -SCHN-), 4.83 (s, 2H, ‑OCH_2_), 3.38 (s, 2H, COCH_2_S-); ^13^C{^1^H}-NMR: δ 35.8 (COCH_2_S), 45.5 (CH_2_), 57.4 (NCHS), 69.1 (CH_2_O-) 107.6 (C-8), 111.0 (C-6), 112.5 (C-3), 113.4 (C-10), 127.2 (C-4, Ar-), 127.8 (C-5), 128.7 (C-3,5, Ar-),128.8 (C-2,6 Ar-), 139.2 (C-1, Ar-), 151.2 (C-9), 155.0 (C-4), 160.3 (C-7), 160.9 (C-2), 166.4 (COCH_2_O), 168.8 (SCH_2_CO-N), 173.3 (CONH-). Found, %: C, 61.3; H, 4.4; N, 6.8; O, 19.5; S, 7.7. C_21_H_18_O_5_N_2_S. Calculated, %: C, 61.45; H, 4.42; N, 6.83; O, 19.49; S, 7.81.

*N-(2-(3-Methoxyphenyl)-4-oxothiazolidin-3-yl)-2-(4-methyl-2-oxo-2H-chromen-7-yloxy)acetamide* (**5b**). m.p. 184 °C, yield 76%; IR: ν_max_ CO lactone: 1,681 cm^−1^, CONH (amide): 1,614 cm^−1^; ^1^H-NMR: 8.62 (s, 1H, NH-), 2.20 (s, 3H), 7.76 (d, *J*H_5.6_ = 10.5 Hz, 1H, H-5), 7.60–7.30 (m, 8H, arom.), 3.67 (s, 3H, OCH_3_), 7.04 (d, *J*H_5.6_ = 10.5 Hz, 1H, H-6), 7.02 (s, 1H, H-8), 6.34 (s, 1H, H-3), 5.92 (s, 1H, NCHS), 4.84 (s, 2H, -OCH_2_), 3.38 (s, 2H, COCH_2_S); ^13^C{^1^H}-NMR: δ 35.7 (COCH_2_S), 45.5 (CH_2_), 57.4(NCHS), 69.10 (CH_2_O-), 107.6 (C-8), 111.0 (C-6), 112.5 (C-3), 113.4 (C-10), 127.0 (C-5), 129.0 (C-3, Ar-), 130.6 (C-6, Ar-), 132.5 (C-4, Ar-), 133.4 (C-1, Ar-), 134.0 (C-2, Ar-), 143.0 (N=CH-), 151.2 (C-9), 155.0 (C-4), 160.3 (C-7), 160.9 (C-2), 166.4 (COCH_2_O), 173.0 (CONH-). Found, %: C, 59.9; H, 4.5; N, 6.4; O, 21.8; S, 7.2. C_22_H_20_O_6_N_2_S. Calculated, %: C, 59.99; H, 4.58; N, 6.36; O, 21.79; S, 7.28.

*2-(4-Methyl-2-oxo-2H-chromen-7-yloxy)-N-(4-oxo-2-(2,3,4-trimethoxyphenyl)thiazolidin-3-yl)acetamide* (**5c**). m.p. 239–241 °C, yield 52%; IR: ν_max_ 1,712 (C=O, lactone), 1,624 (C=O, amide) cm^−1^; ^1^H-NMR: 2.12 (s, 3H), 8.30 (s, 1H, NH-), 7.76 (d, *J*H_5.6_ = 10.4 Hz, 1H, H-5), 7.61–7.30 (m, 6H, arom.), 7.04 (d, *J*H_5.6_ = 10.4 Hz, 1H, H-6), 7.02 (s, 1H, H-8), 6.34 (s, 1H, H-3), 5.92 (s, 1H, NCHS), 4.82 (s, 2H, ‑OCH_2_), 3.75 (s, 9H, 2,3,4 OCH_3_), 4.28 (s, 2H, CH_2_), 3.38 (s, 2H, COCH_2_S); ^13^C{^1^H}-NMR: δ 35.7 (COCH_2_S), 45.5 (CH_2_), 47.4 (NCHS), 69.10 (CH_2_O-), 103.7 (C-3, Ar-), 107.6 (C-8), 108.4 (C-5, Ar-), 110.1 (C-1, Ar-), 111.0 (C-6), 112.5 (C-3), 113.4 (C-10), 127.8 (C-5),131.3 (C-6, Ar-), 151.2 (C-9), 157.2 (C-2, Ar-), 158.2 (C-4), 160.3 (C-7), 160.9 (C-2), 166.4 (CONH),168.8 (NCOCH_2_), 173.3 (CH_2_CONH). Found, %: C, 57.4; H, 4.75; N, 5.7; O, 25.6; S, 6.5. C_24_H_24_O_8_N_2_S. Calculated, %: C, 57.59; H, 4.83; N, 5.60; O, 25.57; S, 6.41.

*N-(2-(4-Bromophenyl)-4-oxothiazolidin-3-yl)-2-(4-methyl-2-oxo-2H-chromen-7-yloxy)acetamide* (**5d**). m.p. 240–241 °C, yield 72%; IR: ν_max_ 1,727 (CO, lactone), 1,616 (C=O, amide) cm^−1^; ^1^H-NMR: 2.14 (s, 3H), 8.22 (s, 1H, NH-), 7.76 (d, *J*H_5.6_ = 10.5 Hz, 1H, H-5), 7.67–7.30 (m, 8H, arom.), 7.04 (d, *J*H_5.6_ = 10.5 Hz, 1H, H-6), 7.02 (s, 1H, H-8), 6.34 (s, 1H, H-3), 5.92 (s, 1H, NCHS), 4.84 (s, 2H, -OCH_2_), 3.38 (s, 2H, COCH_2_S); ^13^C{^1^H}-NMR: δ 35.7 (COCH_2_S), 45.5 (CH_2_), 57.4 (NCHS), 69.10 (CH_2_O-), 107.6 (C-8), 111.0 (C-6), 112.5 (C-3), 113.4 (C-10), 127.3 (C-6, Ar-), 127.8 (C-5), 129.3 (C-2, Ar-), 130.3 (C-5, Ar-), 131.2 (C-4, Ar-), 135.2 (C-1, Ar-), 134.4 (C-3, Ar-), 143.0 (N=CH-), 151.2 (C-9), 155.0 (C-4), 160.3 (C-7), 160.9 (C-2), 173.0 (COCH_2_O), 173.0 (CONH-). Found, %: C, 51.6; H, 3.4; Br, 16.4; N, 5.75; O, 16.4; S, 6.7. C_21_H_17_O_5_N_2_BrS. Calculated, %: C, 51.54; H, 3.50; Br, 16.33; N, 5.72; O, 16.35; S, 6.55.

### 3.2. DPPH Test

The DPPH test aims to measure the capacity of the compounds for scavenging the stable free radical 2,2-diphenyl-1-picrylhydrazyl (DPPH^.^) by donation of hydrogen atom or an electron [23] If the compounds have the capacity to scavenge the DPPH free radical, the initial blue/purple solution will change to a yellow colour due to the formation of diphenylpicrylhydrazine. Scavenging capacity was read spectrophotometrically by monitoring the decrease of the absorbance at 517 nm. Lower absorbance of the reaction mixture indicates higher free radical scavenging activity.

The percent DPPH scavenging effect was calculated using the following equation: 



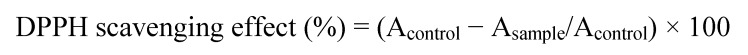


where A_control_ is the absorbance of the control reaction were the sample is replaced by 500 µL ethanol. Tests were carried out in triplicate.

### 3.3. ABTS Radical Cation Decolourization Assay

The potential of *N*-(2-aryl-4-oxo-thiazolidin-3-yl)-2-(4-(2-aryl-4-oxo-thiazolidin-3-ylcarbamoyl)-methyl)-2-oxo-*2H*-chromen-7-yloxy)-acetamides **5a**–**d** to scavenge free radicals was also assessed by checking their ability to quench ABTS^.^ depicted by the concentration-dependent decolourization of ABTS^.^. ABTS radical-scavenging activity of **5a**–**d** was determined according to Güven *et al.* [24]. The ABTS^.^ cation radical was produced by the reaction between 5 mL of 14 mM ABTS solution and 5 mL of 4.9 mM potassium persulfate (K_2_S_2_O_8_) solution, stored in the dark at room temperature for 16 h. Before use, this solution was diluted with ethanol to get an absorbance of 0.700 ± 0.020 at 734 nm. In a final volume of 1 mL, the reaction mixture comprised 950 µL of ABTS^.^ solution and 50 µL of **5a**–**d** at various concentrations. The reaction mixture was homogenized and its absorbance was recorded at 734 nm. Ethanol blanks were run in each assay, and all measurements were done after at least 6 min. Similarly, the reaction mixture of standard group was obtained by mixing 950 µL of ABTS^.^ solution and 50 µL of Trolox. As for the antiradical activity, ABTS scavenging ability was expressed as IC_50_ (µg/mL). The inhibition percentage of ABTS radical was calculated using the following formula:



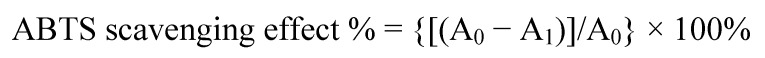


where A_0_ is the absorbance of the control at 30 min, and A1 is the absorbance of the sample at 30 min. All samples were analyzed in triplicate.

### 3.4. Antibacterial Activity

Antimicrobial activity of the purified compounds was evaluated by means of agar-well diffusion assay according to Güven *et al.* [25] with some modifications. The nutrient agar broth prepared by the usual method, was inoculated aseptically with 0.5 mL of 24 h old subculture of *S. aureus* ATCC 2353, *B. megaterium*, *P. vulgaris*, and *E. coli ATCC1225* in separate conical flasks at 40–50 °C and mixed well by gentle shaking. About 25 mL of the contents of the flask were poured and evenly spread in petridish (90 mm in diameter) and allowed to set for two h. The cups (10 mm in diameter) were formed by the help of borer in agar medium and filled with 0.04 mL (40 μg/mL) solution of sample in DMF. The plates were incubated at 37 °C for 24 h and the control was also maintained with 0.04 mL of DMF in similar manner and the zones of inhibition of the bacterial growth were measured in millimetre

## 4. Conclusions

A series of new coumarin-based thiazolidinone compounds were successfully synthesized, characterized and tested for their antibacterial and antioxidant activities. Most of the synthesized compounds are more active against *E. coli* and *S. aureus* than standard references. Some of the compounds were found equipotent to ampicillin, but less active than other used standard drugs
